# Superior outcomes of nodal metastases compared to visceral sites in oligometastatic colorectal cancer treated with stereotactic ablative radiotherapy

**DOI:** 10.1016/j.radonc.2020.08.012

**Published:** 2020-10

**Authors:** Sean M. O'Cathail, Thomas Smith, Rob Owens, Anthi Zeniou, Yatman Tsang, Daniel L.P. Holyoake, Louise Murray, Mark Harrison, Maria A. Hawkins

**Affiliations:** a Institute of Cancer Sciences, University of Glasgow, UK; b Mount Vernon Cancer Centre, UK; c Oxford University NHS Foundation Trust, UK; d Leeds Cancer Centre, UK; e Norwich and Norfolk University NHS Foundation Trust, UK; f Medical Physics and Biomedical Engineering, University College London and University College London Hospitals NHS Foundation Trust, UK

**Keywords:** Colorectal, Stereotactic, SBRT, Oligometastatic, Lymph node

## Abstract

**Background:**

Stereotactic ablative radiotherapy (SBRT) is a radical option for oligometastatic colorectal cancer (CRC) patients, but most data relate to visceral metastases.

**Methods:**

A prospective, multi-centre database of CRC patients treated with SBRT was interrogated. Inclusion criteria were ECOG PS 0–2, ≤3 sites of disease, a disease free interval of >6 months unless synchronous liver metastases. Primary endpoints were local control (LC), progression free survival (PFS) and overall survival (OS).

**Results:**

163 patients (172 metastases) were analysed. The median FU was 16 months (IQR 12.2–22.85). The LC at 1 year was 83.8% (CI 76.4%−91.9%) with a PFS of 55% (CI 47%−64.7%) respectively. LC at 1 year was 90% (CI 83%−99%) for nodal metastases (NM), 75% (63%−90%) for visceral metastases (VM). NM had improved median PFS (9 vs 19 months) [HR 0.6, CI 0.38–0.94, *p* = 0.032] and median OS (32 months vs not reached) [HR 0.28, CI 0.18–0.7, *p* = 0.0062] than VM, regardless of whether the NM were located inside or outside the pelvis. On multivariate analysis, NM and ECOG PS 0 were significant good prognostic factors. An exploratory analysis suggests KRAS WT is also a good prognostic factor.

**Conclusion:**

Nodal site is an important prognostic determinant of SBRT that should incorporated into patient selection. We hypothesise this may have an immunoediting basis.

## Highlights

• SBRT for CRC results in excellent local control rates for nodal metastases.
• Median PFS for NM was 19 months versus 9 months for VM.
• Nodal site was significant prognostic factor on multivariate analysis for PFS/OS.
• We hypothesise an immunoediting basis for the improved outcomes of NM.

Approximately 20% of colorectal cancer (CRC) patients present with stage IV disease [1]. Of those that present at an early stage and are treated radically, 20–30% will go on to develop metastatic disease [2], [3]. Systemic therapy is the main treatment for metastases given the proven survival benefit, however metastasis directed therapy is increasingly being used to manage metastatic deposits in an attempt to achieve long term benefit [4]. Aggressive management of patients who have unresectable liver disease at the time of diagnosis [5] using radiofrequency ablation (RFA) has demonstrated an overall survival benefit versus standard of care [6]. The recent randomised phase II SABR-COMET trial demonstrated a survival benefit from the addition of stereotactic ablative body radiotherapy (SBRT) in oligometastatic disease at extra-cranial sites [7], where almost 20% of the patients had CRC. These data suggest that CRC patients can derive significant benefit in limited metastatic disease.

The oligometastatic state lies on a spectrum between localised and disseminated disease [8]. A concrete definition is lacking with the most common criteria being the number and location of radiologically identifiable metastases. The ESMO consensus guidelines for the management of patients with metastatic colorectal cancer, defines oligometastases as five or sometimes more metastases at two or three sites, primarily visceral and lymph nodes [9]. Data from surgical cohorts in CRC demonstrate an improved 5-year OS for patients with 1–3 resectable metastases, compared to 4–6, or more than 6, respectively [10], suggesting burden of disease is important. More recently there have been reports focusing on a specific primary cancer type or a specific treated metastatic site [11], [12], [13], [14], [15].

Visceral metastases (VM) are the most common metastases from CRC that are treated with SBRT [16] and much of the literature to date has focussed on either liver or lung metastases [17]. In the SABR-COMET trial [7] most patients had visceral metastases, while only 3 (6%) of metastases treated with SBRT were in lymph nodes. There are little data available on the outcomes of lymph node only oligometastases in colorectal cancer treated with SBRT. The treatment options for these patients are limited to systemic therapy, as RFA and surgery are less commonly performed, and there is some debate about whether or not the entire nodal chain should be treated. Furthermore, the mechanisms of spread for visceral and nodal oligometastases differ which may have implication for outcomes.

We analysed a prospectively collected, multicentre cohort of oligometastatic CRC patients treated with SBRT to identify differences in outcomes between treated visceral metastases (VM) and lymph node metastases (NM) at oligometastatic sites.

## Methods

### Study population

Patients with CRC were identified from a prospectively collected register of patients [18] diagnosed with colorectal cancer treated across three UK sites (Oxford, Mount Vernon, Leeds). Key eligibility criteria were: confirmed histological diagnosis, ECOG PS 0–2, ≤3 sites of disease and no more than 2 organ systems, no brain metastases, primary tumour resected with a disease free interval of >6 months (synchronous presentations were permitted for liver metastases) as identified on multimodality imaging (CT, PET and MRI as appropriate), adequate organ function and no systemic treatment for 28 days or planned systemic treatment after SBRT. All nodes were confirmed as isolated through review of serial imaging. Patients with less than 3 months of follow were excluded. All metastatic lesions were treated, where there was more than one. All patients consented to collection of data as part of enrolment in the SBRT treatment program which had received ethical approval (North East – York Research Ethics Committee REC reference: 16_NE_0285).

## Techniques of radiotherapy

All patients were scanned with helical CT using ≤5 mm interval. Gross tumour volume (GTV) was outlined and clinical target volume (CTV) was equal to GTV for all lesions except liver metastases where, a 5 mm margin in all directions was applied added to GTV to define CTV. Radiotherapy planning CT images were co-registered with diagnostic radiology at the treating oncologist’s discretion. Where disease sites were subject to internal movement (such as lung or liver), patients were planned using 4D-CT scan. Abdominal compression or fiducial tracking [19] was used for abdominal motion management. A margin of 3–5 mm, depending on disease site and dimensions, was added to GTV/ CTV to obtain the planning target volume (PTV). Details of radiation doses, which varied according to tumour sites, are provided in Supplementary Table 1. An *α*/*β* ratio of 10 was used for biologically effective dose (BED) calculations. KRAS mutation status of the primary tumour was collected by retrospective review of pathology reports. All mutations were activating driver mutations and detected by next generation sequencing of the primary tumour using a targeted gene panel.

## Response assessment

First evaluation was planned 3 months after the end of the SBRT and then every 3 months for the first year and every 6 months from the second to the fifth year. Follow-up visits included clinical evaluation and diagnostic imaging (CT, MRI or PET scan) at treating physician’s discretion. End points of the present study were local control (LC), defined as absence of progression inside the SBRT treated volume; locoregional progression (LRP), defined as progression outside the treated volume in an adjacent nodal station/chain or within the same organ (liver/lung) and distant progression (DP), as metastasis within another organ system or anatomically remote from the treated lesion. Toxicity data was collected as part of the overall treatment program and is publically available [20].

### Statistical analysis

All outcomes were calculated from date of SBRT treatment. Time to any progression (LC/LRP/DP) was defined as PFS and overall survival (OS) treatment to either death or censoring. Univariate analysis was performed with the log-rank test, and Cox proportional hazards regression was used to estimate hazard ratios (HR). The primary endpoints were PFS and OS. Multivariable stepwise cox regression analysis was performed to evaluate the association between clinical factors and survival, with a significance level of *p* < 0.05. Survival analysis was performed using Cox regression models and Kaplan Meier estimates with log rank testing. Median follow-up was ascertained by reverse-censoring method. Patients without the event of interest were censored at the time last known to be event-free. All statistical analysis was performed using R statistical software [21].

## Results

A total of 184 patients were treated between September 2015 and October 2018. Nine were excluded as the treated site was intracranial and 12 excluded due to inadequate or missing follow up. The final cohort was 163 patients with 172 treated lesions. The median follow up was 16 months (IQR 12.2–22.85). The cohort characteristics are summarised in Table 1. Toxicity was consistent with published series and is available elsewhere, with no deaths due to SBRT [20]. The median BED_10_ for the all sites was 79.2 Gy. All lesions received the prescribed dose. Only 23% of patients had not received prior systemic therapy prior to SBRT. KRAS mutation status was available for 64 (39%) of patients.

**Table 1:** Cohort characteristics.

Variable	*N* = 163	%
Age	69 (Range 36–91)	
Gender		
Male	90	55
Female	73	45
ECOG		
0	99	61
1	52	32
2	5	3
Unknown	7	4
Primary site		
Rectum	81	50
Colon	82	50
Treated site		
Liver	38	23
Lymph Node	86	53
Lung	34	21
Other*	5	3
Median BED _10_ (across all sites)	79.2 Gy (IQR 48–105)	
KRAS status		
Wild type	45	28
Mutant	19	12
Not tested	99	61
GTV	9.725 cm^3^ (Range 2.03–39.2)	
Metachronous	135	83
Synchronous	28	17
Lines of chemotherapy		
0	38	23
1	86	53
2	34	21
3	2	1
Number of metastasesǂ		
1	151	93
2	10	6
3	2	1

*Tail of pancreas, left flank, pancreas bed, spine and penile bulb.

ǂ >1 metastases treated as a single GTV are considered as isolated metastases.

The 1 and 2 year local control rate for the whole cohort was 83.8% (CI 76.4%−91.9%) and 77.4% (CI 67.9%−88.2%) respectively. However, the 1 year local control rate varied significantly different according to treated site; 58% for liver (36.7%−92.7%), 90% for lymph nodes (82.9%−99%) and 92% for lung (80.3%−100%).

**Fig. 1:**
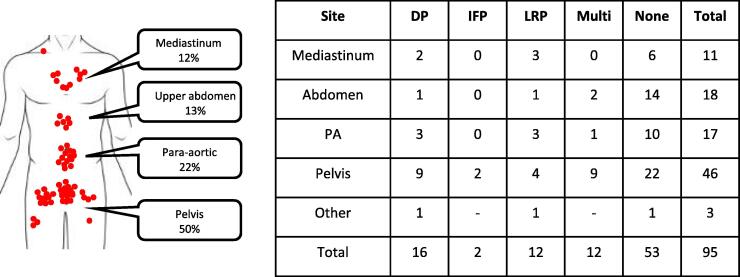
Body diagram showing the anatomical sites of metastases with an associated count grid indicating the outcome (distant progression [DP], in field progression [IFP], locoregional progression [LRP]).

In total, 86 patients with 95 lymph nodes metastases were treated. These were mapped to four anatomical locations: mediastinum, upper abdomen, para-aortic nodes and pelvic/inguinal nodes [Fig. 1]. 53 out of 95 (56%) of nodes never progressed. Of the remaining 42, only 2 progressed in-field and 12/42 (28%) progressed at multiple sites.

**Fig. 2:**
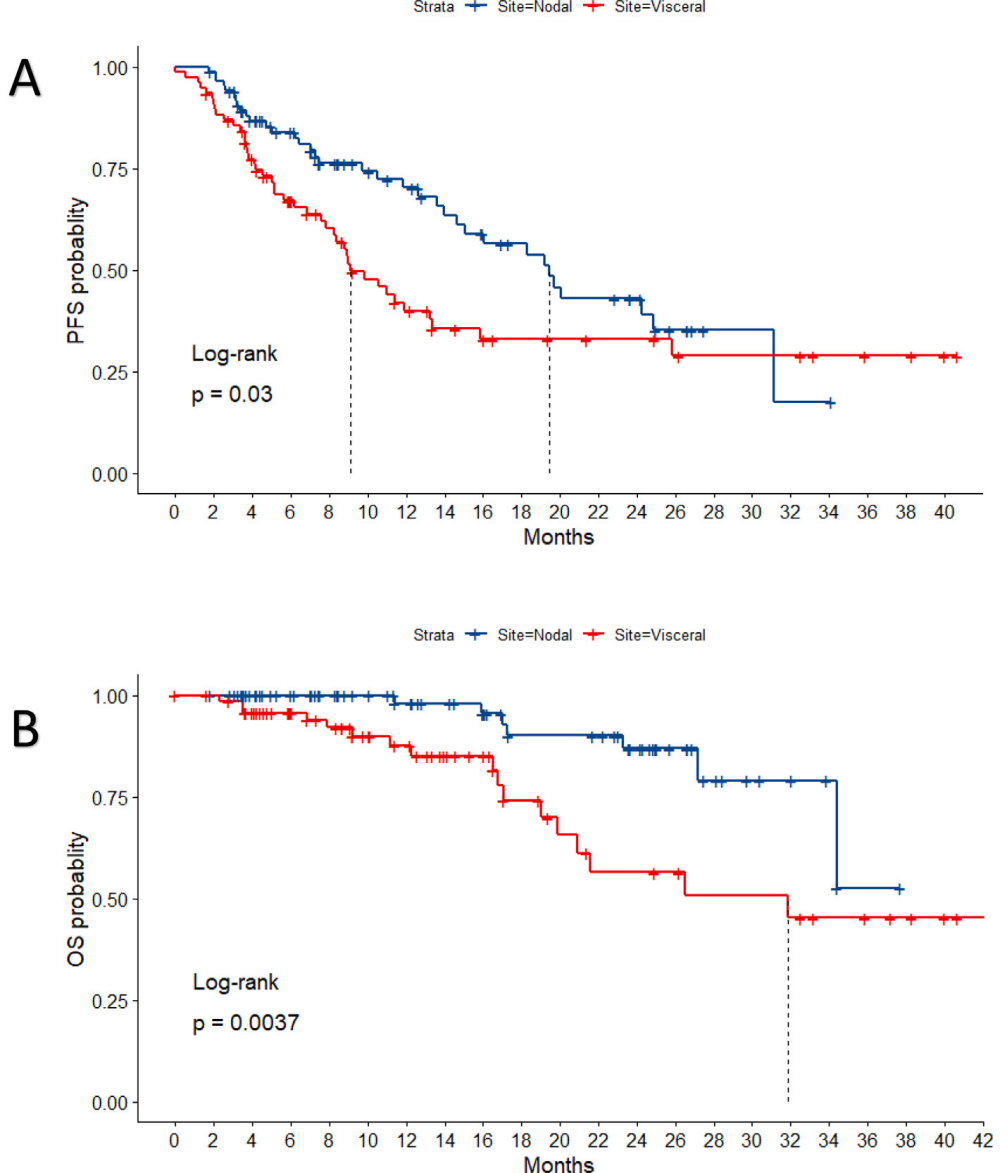
Kaplan Meier plots with associated risk tables of progression free survival (A) and overall survival (B) outcomes for visceral (red) and nodal (blue) metastases.

At 1 year and 2 years respectively, PFS for the whole cohort was 55% (CI 47%−64.7%) and 37.6% (CI 29%−48.8%) while OS was 93% (88.6%−98%) and 74% (CI 64.5%−85.4%). Median PFS for the whole cohort was 13.9 months, with median OS not reached. VM (liver, lung & bone) had a worse median PFS (9 months vs 19 months) and worse median OS (32 months vs not reached) than nodal metastatic sites, reflected in a statistically significant difference by Cox regression for PFS [HR 0.6, 95% 0.38–0.94, *p* = 0.032] and OS [HR 0.28, 95% 0.18–0.7, *p* = 0.0062] [Fig. 2]. On univariate analysis there was no significant in PFS difference for ECOG PS, primary site or synchronous/metachronous disease at presentation (Table 2). Patients in receipt of chemotherapy (adjuvant or metastatic) prior to SBRT had an increased hazard for progression [HR 1.93, C.I 1.08–3.45; *p* = 0.027]. On univariate analysis for OS, ECOG PS 1 or 2 were associated with an increased risk of death, relative to PS 0, but only the former was statistically significant with an overall low number (5) of PS 2 patients [Table 2]. Patients who had previously received systemic chemotherapy prior to SBRT had an increased hazard for progression [HR 1.93, CI 1.08–3.45; *p* = 0.027] and increased hazard for death, with a trend towards statistical significance.

**Table 2:** Univariate analysis for local control, progression free survival and overall survival.

	LC		PFS		OS	
Variable	HR (CI)	*p* value	HR (CI)	*p* value	HR (CI)	*p* value
ECOG PS						
0			*Reference*			
1	1.11 (0.43–2.86)	0.831	1.02 (0.63–1.65)	0.932	2.75 (1.13–6.68)	0.025*
2	2.28 (0.29–17.75)	0.43	0.51 (0.07–3.71)	0.506	4.85 (0.6–39.14)	0.138
Primary site						
Rectum (ref Colon)	0.88 (0.37–2.12)	0.779	1.0 (0.64–1.55)	0.984	0.7 (0.31–1.57)	0.382
Prior chemotherapy (ref no chemotherapy)	1.71 (0.57–5.15)	0.337	1.93 (1.08–3.45)	0.027*	3.18 (0.94–10.72)	0.063
Synchronous presentation (ref metachronous)	0.88 (0.26–3.02)	0.842	1.61 (0.96–2.71)	0.070	1.32 (0.49–3.55)	0.579
Lymph node site (ref visceral site)	0.6 (0.25–1.46)	0.262	0.61 (0.39–0.96)	0.032*	0.29 (0.12–0.7)	0.006*

*Denotes statistically significant at the 0.05 level.

Significant factors on univariate testing were included in a multivariate analysis for PFS and OS [Table 2], where VM remained significantly associated with poor outcomes. Inclusion of local control in a OS multivariate cox model showed that poor local control and an ECOG PS 1/2 were significantly associated with worse overall survival (Supplementary Table 2). NM site was associated with an improved OS outcome [HR 0.37, CI 0.14–0.95, *p* = 0.038].

**Fig. 3:**
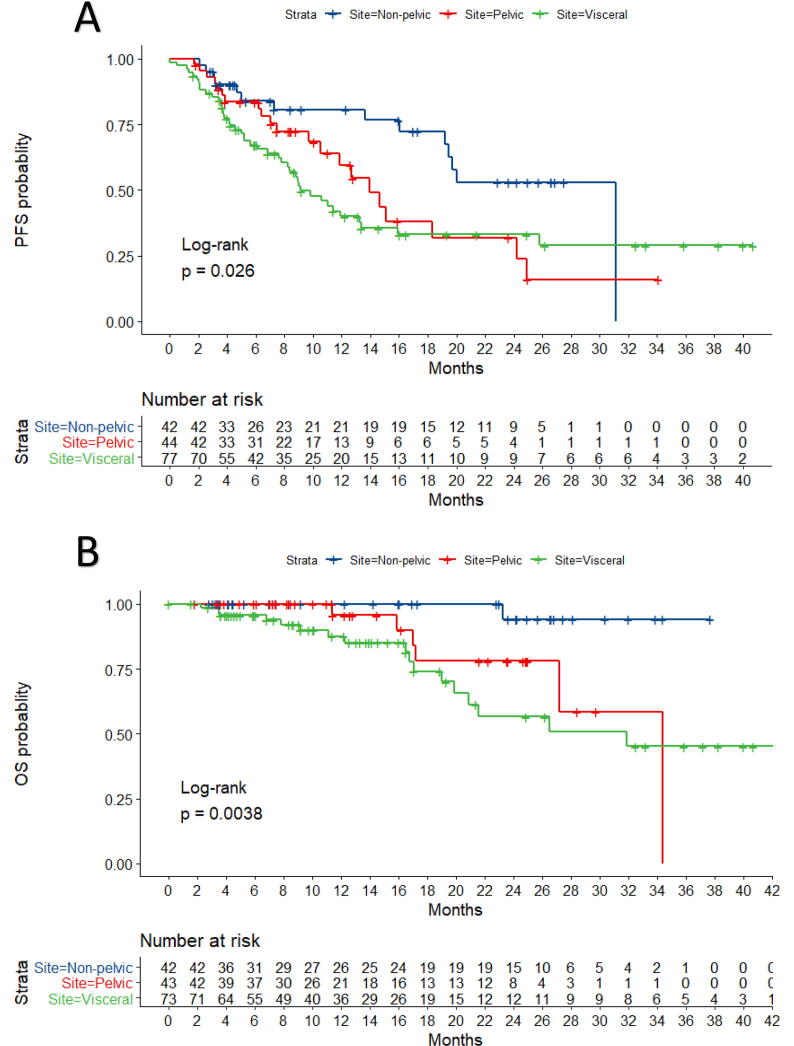
Kaplan Meier plots with associated risk tables of progression free survival (A) and overall survival (B) outcomes for pelvic NM, distant NM (outside the pelvis) and visceral metastases.

To understand if the improved outcomes of NM was due to the large proportion of pelvic LN (50%), these were compared to distant, extra-pelvic NM sites and VM. On Cox regression analysis, relative to extra-pelvic LN, VM had an inferior PFS [HR 2.24, C.I 1.23–4.17; *p* = 0.008] and inferior OS [13.9, C.I 1.85–105.6; *p* = 0.01] but pelvic LN did not have significantly worse PFS [HR 1.86, C.I 0.94–3.68; *p* = 0.074] or OS [HR 8.15, C.I 0.97–67.85; *p* = 0.052] [Fig. 3].

The effect of KRAS mutation status was explored in the subgroup of patients for whom mutation testing of the primary tumour was available (*N* = 64), 30% of which were KRAS mutant. Other activating mutations, such as BRAF, were less common as expected [22], and not detected in sufficient numbers for analysis. There was no significant difference in the distribution of oligometastatic sites (liver, node, lung, bone) by KRAS mutation (Fisher’s exact test, *p* = 0.346). There was no difference in local control rates between KRAS wild type and mutant cases (log rank *p* = 0.63) [Fig. 3A]. KRAS wild type was associated with improved PFS [HR 0.42, 95% CI 0.2–0.87; *p* = 0.02] and OS [HR 0.2, 95% CI 0.05–0.76; *p* = 0.02] [Supplementary Fig. 1]. The median PFS for wild type was 13 months versus 7 for mutant patients. On multivariate Cox regression analysis, nodal metastases and KRAS wild type remained significant prognostic factors [Nodal HR 0.09, 95% CI 0.02–0.4, *p* = 0.002; KRAS wild type HR 0.1, 95% 0.01–0.5, *p* = 0.007].

## Discussion

In this prospectively collected, multicentre cohort study we showed patients having SBRT for nodal oligometastases enjoy better survival outcomes, relative to those treated for visceral metastases. The cohort was accrued over a short period of time (3 years) and had a LC at 1 and 2 years of 83.8% and 77.4%, respectively. LC in liver metastases appeared to be worse compared to other sites within the cohort and consistent with a systematic review of SBRT in CRC [23], which estimated wide variation local control rates for liver metastases of between 50%-100% at 1-year and 32%-91% at 2 years. Previous data had suggested that CRC patients with oligometastatic disease to liver have worse outcomes compared to other disease types treated with SBRT [13], [24]. Radiosensitivity among liver metastases from CRC is heterogeneous compared to other sites [25] and this may account for the varying local controls rates [23].

A recent large single-centre CRC demonstrated 1-year local control of 95% and 3-year rate of >70% for the whole cohort [11]. Although there was no difference in LC between lung vs non-lung metastases, they did not present the LC rates by site and had low numbers of nodal sites (12.4%). Factors which negatively influenced OS in multivariate analysis were non-lung sites [HR 1.97 (1.30–2.99), *p* = 0.02], CTV > 30 mm [HR 1.73 (1.18–2.55), *p* = 0.03], systemic therapy before SBRT [HR 1.61 (1.01–2.57), *p* = 0.023] and poor local control [HR 1.59 (1.04–2.43), *p* = 0.007]. Similar findings had been reported in a multi – tumour cohort where prior systemic therapy resulted in worse LC [13]. A consistent interpretation is that achieving good local control of treated sites can lead to improved survival outcomes. Although local control, strictly speaking, is an outcome variable as opposed to pre-treatment variable, and thus has no use in selecting patients for SBRT, such analyses are common in SBRT cohorts. It should be noted however that assessment of local control could vary between reporting radiologists in a multi-institutional study, imaging modalities and anatomical sites, particularly liver lesions. Even though all participating centres were high volume, experienced, accredited SBRT institutions this is a potential weakness in our LC estimates.

The majority (56%) of nodal metastases never progressed in field during follow up. Of the 42 that did progress, 12 patients had LRP only and 28% had LRP and multi-site progression while 38% had distant disease. The pattern of relapse post SBRT justifies considering local tumoricidal treatments only to isolated nodal disease. Introduction of CTV around NM may decrease LRP but would increase toxicity. The excellent local control achieved with SBRT in nodal disease translated into an improved time to progression (19 vs 9 months) and sustained into an OS benefit. Conversely, worse local control as seen in the liver metastases, was associated with worse OS in the multivariate analysis [HR 3.3 (95% CI 1.35 – 8.78), *p* = 0.016]. Given that 50% of visceral metastases had progressed by 9 months suggests that in such patients SBRT and systemic therapy could be better therapeutic approach, analogous to liver resections.

KRAS mutation has recently been shown to be a prognostic biomarker of worse survival outcomes in metastatic colorectal cancer in a large meta-analysis of first line randomised chemotherapy trials [26], an analogous situation to those referred for SBRT. Kinj et al found that KRAS mutation was associated with inferior metastasis free survival, but not OS, following SBRT in lung metastases [14]. In a randomised phase II trial of proton therapy for liver metastases KRAS mutants and TP53 mutants had worse local control than wild type patients [24]. A recent comprehensive study of tumour mutation status in a multisite cohort [27] demonstrated similar findings. Interestingly, although only 10% of their cohort, NM had 100% LC at 2 years. We suggest that KRAS mutation is a relevant prognostic factor in oligometastatic CRC and be incorporated as a stratifying factor into future SBRT studies.

Our study has limitations. Our median follow up is shorter than some published datasets [11], [28], [29], but not all [30], in part due to data collection permissions. However, the significant majority of relapse events after surgery occur in the first 2–3 years [2]. Our cohort had already accrued 6 months DFS prior to entry in the SBRT program, in addition to the median follow up which was calculated from date of SBRT. Our cohort represents a very clear subset of the recent ESTRO/EORTC OMD classifier [31] – metastatic oligorecurrence – which represents 83% [Table 1] and should be interpreted as such. We did not have histological and molecular mutation confirmation of every treated site and thus inferred KRAS status. KRAS status of the primary tumour shows high concordance with mutation status in tissue sampled from metastases [32], [33], [34] however. Given the small sample numbers of known KRAS patients, the analysis could be subject overfitting in MVA and is considered hypothesis generating.

Although a variety of radiotherapy doses were used, leading to range of BED, each site was treated consistently with the same dose. The schedules used are equivalent to those mandated in the SABR-COMET trial [7]. BED_max_ has previously been attributed to improved LC in liver metastases [12], [13]. An analysis of dose effect on outcomes is not possible as it is confounded by treated site. Here, NM had the best LC despite the lowest prescription dose (BED_10_ 60–93.3 Gy) suggesting more fundamental biological differences in radiation response between sites.

One potential working hypothesis for the observed differences are different routes of spread, with visceral metastases spreading haematologically and nodal metastases through the lymphatic system. The ability of the immune system to influence a cancer’s clinical course – “cancer immunoediting” - is marked by three distinct phases: elimination, equilibrium, and escape [35]. The clinical existence of oligometastases suggests that these tumours have escaped cancer immunoediting. LN are historically viewed as production sites for antigen-specific (adaptive) effector cells but they also contain a spatially co-ordinated diverse multicellular network of lymphoid cells (innate) that can rapidly generate a cytokine response [36]. Radiotherapy engages both the adaptive and innate immune system to convert the irradiated tumour into an ‘in-situ vaccine’ that elicits a tumour specific T-cell response [37]. In doing so, radiotherapy can assist recalibration of the immunoediting process, switching escape back to elimination and equilibrium. Once an oligometastatic site is treated, the ‘vaccinated’ individual may have the immune memory capacity to prevent (elimination) or defer (equilibrium) the development of synchronous disease sites.

In support of this theory, a recent study by Pitroda et al, of integrated molecular analysis of CRC metastases, an immune enriched subtype developed limited numbers of clinically evident synchronous metastases and was associated with improved survival outcomes [38]. These data would be consistent with the immunoediting hypothesis. Furthermore, they demonstrated that increased KRAS signalling was associated with worse survival outcomes, consistent with our data.

Treatment options for metastatic CRC are slow to progress compared to other common cancers, with cytotoxic chemotherapy still the mainstay of treatment. SBRT is an excellent tool that offers a radical, potentially curative, option to patients with limited disease spread. However, optimum selection of patients and sequencing of therapies to maximise benefit has yet to be clarified. The current study represents an important step forward in highlighting the need for biological selection of patients for SBRT, in addition to known clinical factors. A better understanding of the local and circulating immune response generated by SBRT is needed to fully explain the varying outcomes seen in this and other studies of oligometastatic CRC.

## Conflict of interest

The authors have no relevant disclosures

## Funding

SMO’C is a CRUK funded clinical senior lecturer at the Institute of Cancer Sciences (grant number CAN-RES-UK (C7932/A25142). MAH is supported by funding from the NIHR Biomedical Research Centre at University College London Hospitals NHS Foundation Trust. LM is currently funded by 10.13039/501100002653Yorkshire Cancer Research (award number L389LM).

## Appendix A Supplementary data

Supplementary data 1 Kaplan Meier plots for clinical outcomes of KRAS wild type compared to KRAS mutant tumours [*N* = 64]. There was no difference in local control (A). Progression free survival (B) and overall survival (C) were significantly improved for KRAS wild type patients with a median difference of 4 months and 17 months respectively.
