# Supplementary figures and images for: Superior outcomes of nodal metastases compared to visceral sites in oligometastatic colorectal cancer treated with stereotactic ablative radiotherapy
Source: Radiother Oncol. 2020 Oct;151:280–6. doi: 10.1016/j.radonc.2020.08.012 (PMC7689579; doi:10.1016/j.radonc.2020.08.012)

## Slide 1
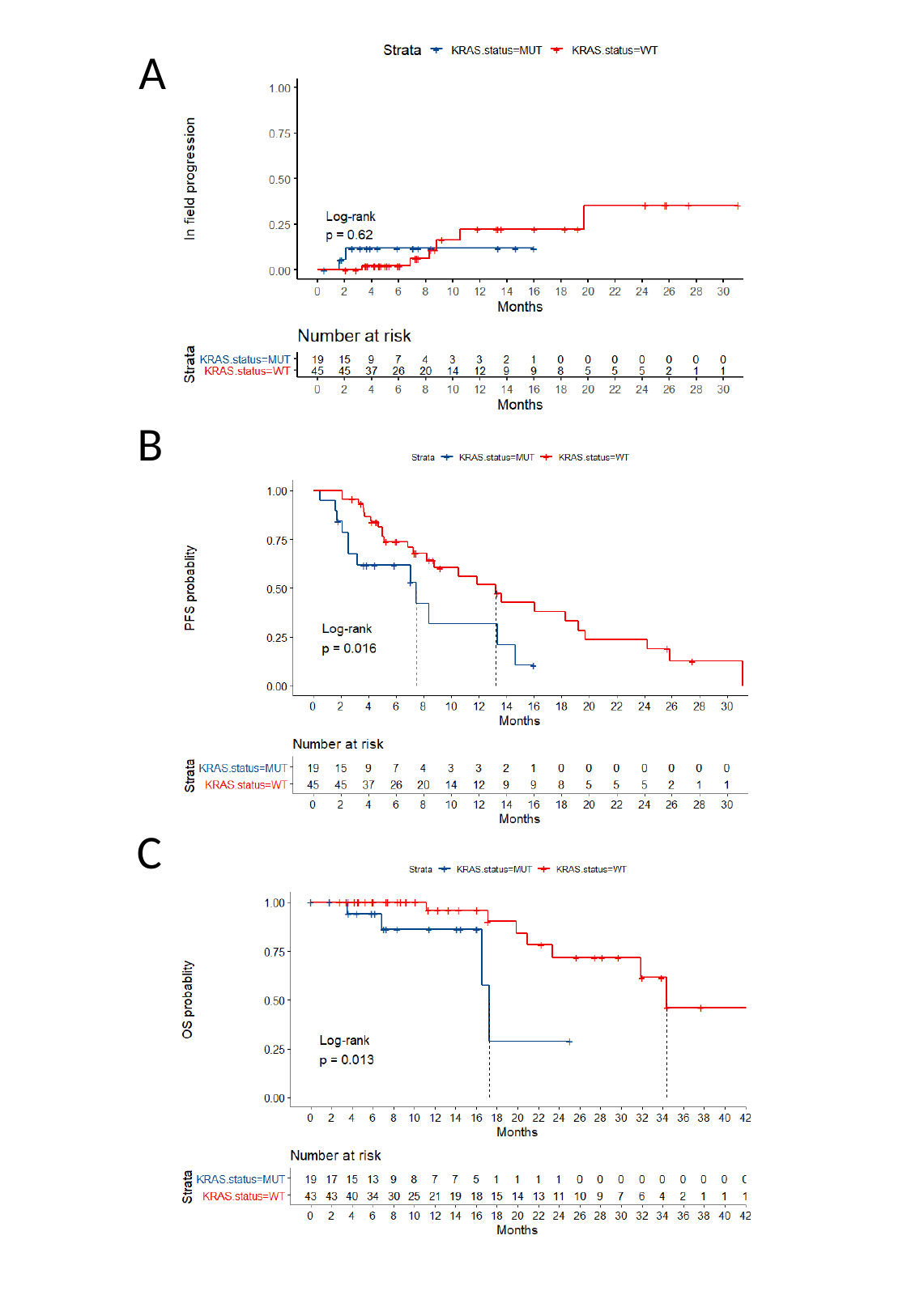

A
B
C

Supplement: Supplementary data 1 — Kaplan Meier plots for clinical outcomes of KRAS wild type compared to KRAS mutant tumours [N = 64]. There was no difference in local control (A). Progression free survival (B) and overall survival (C) were significantly improved for KRAS wild type patients with a median difference of 4 months and 17 months respectively. [file mmc1.pptx]
