# Supplementary material for: Superior outcomes of nodal metastases compared to visceral sites in oligometastatic colorectal cancer treated with stereotactic ablative radiotherapy
Source: Radiother Oncol. 2020 Oct;151:280–6. doi: 10.1016/j.radonc.2020.08.012 (PMC7689579; doi:10.1016/j.radonc.2020.08.012)
Supplement: Supplementary data 2 [file mmc2.docx]

Supplementary Table 1. Dose prescriptions and BED_10_ calculations using an alpha/beta ratio of 10.

|  | Lung | Liver | Lymph nodes | Bone |
| --- | --- | --- | --- | --- |
|  |  |  |  |  |
| Fractionation (BED_10)_ | 54Gy/3  (151.2 Gy) | 50Gy/5  (100 Gy) | 30Gy/3  (60 Gy) | 30Gy/3  (60Gy) |
|  | 60Gy/5  (132 Gy) |  | 36Gy/3  (79.2 Gy) |  |
|  | 60Gy/8  (105 Gy) |  | 40Gy/3  (93.3 Gy) |  |

* All treatments were given on alternate day basis
